# Bioequivalence study of ipratropium bromide inhalation aerosol using PBPK modelling

**DOI:** 10.3389/fmed.2023.1056318

**Published:** 2023-02-07

**Authors:** Jisheng Zhang, Keheng Wu, Bo Liu, Shuguang Hou, Xue Li, Xiang Ye, Jack Liu, Qing He

**Affiliations:** ^1^Wuxi People’s Hospital Affiliated with Nanjing Medical University, Wuxi, Jiangsu, China; ^2^Yinghan Pharmaceutical Technology (Shanghai) Co., Ltd., Shanghai, China; ^3^School of Chemical Engineering and Pharmacy, Wuhan Institute of Technology, Wuhan, China; ^4^Sichuan Purity Medical Technology Co., Ltd., Sichuan, China

**Keywords:** ipratropium bromide, aerodynamic particle size distribution, *in vitro* and *in vivo* correlation, physiologically based pharmacokinetic model, B^2^O simulator

## Abstract

**Aims:**

Systemic pharmacokinetic (PK) studies can reflect the overall exposure of orally inhaled drug Products (OIDPs) in the blood after inhalation into the lung and can be used to evaluate the bioequivalence of test and reference products. The aim of this article is: (1) to study the PK characteristics and bioequivalence of ipratropium bromide (IB) inhalation aerosol, reference and test products in healthy Chinese subjects; (2) to establish a physiologically based pharmacokinetic (PBPK) model and verify the accuracy of the model in predicting bioequivalence; (3) attempt to use the model to predict the regional distribution of particles in the lung after inhalation, and discuss the effect of gastrointestinal drug absorption of IB on systemic exposure.

**Methods:**

The study involved two clinical studies. Clinical study-1 (registration number: CTR20201284) was used with non-clinical data to construct and validate a PBPK model in the B^2^O simulator, a web-based virtual drug development platform. This model assessed different test and reference products’ bioequivalence. Results were compared to a second clinical study (Clinical study-2: registration number CTR20202291). The particles’ regional distribution in the lung and the gastrointestinal absorption effect on systemic exposure were discussed based on the simulation results.

**Results:**

The established PBPK model successfully simulated the *in vivo* PK characteristics of IB inhalation aerosol, with *r*^2^ close to 1. Gastrointestinal absorption had a negligible effect on systemic exposure. Particles accumulated in the alveolar area were cleared within an hour, followed by particles in the bronchioles and bronchi.

**Conclusion:**

This model provided a reliable method for exploring the correlation between *in vitro* and *in vivo* PK studies of IB inhalation aerosols. According to the simulation results, the test and reference products were bioequivalent.

**GRAPHICAL ABSTRACT:**
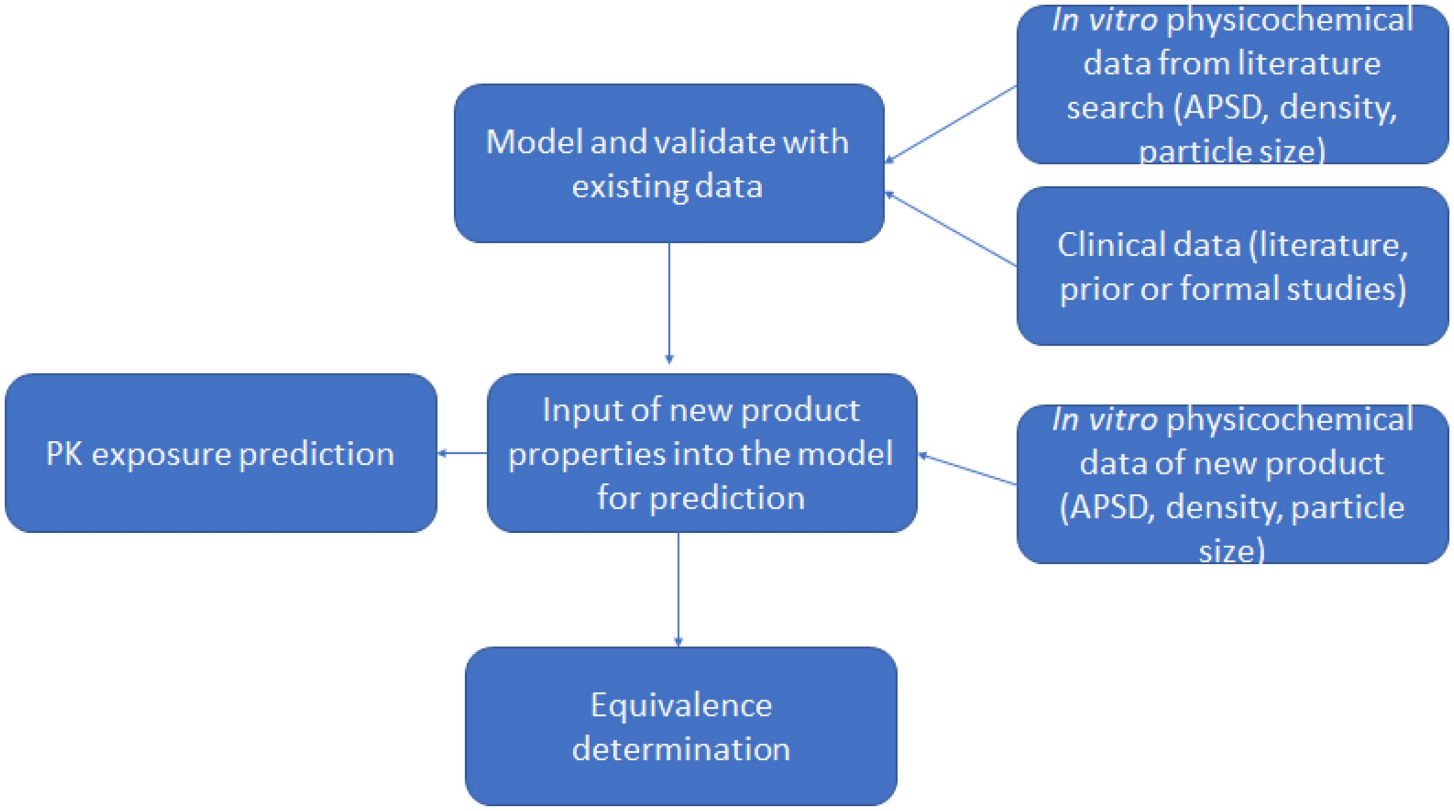


## 1. Introduction

Bronchial asthma and Chronic Obstructive Pulmonary Disease (COPD) are major chronic inflammatory diseases of the respiratory system, bringing severe public health problems to countries worldwide (1, 2). According to the population estimates in China in 2015, among adults aged 20 and above, there were 45.7 and 99.9 million patients with bronchial asthma and COPD, respectively, with an overall prevalence of 4.2 and 8.6%, respectively, in people aged 40 and over, the prevalence of COPD is as high as 13.7% (3, 4). Orally Inhaled Drug Products (OIDPs) are the first choice for the prevention and treatment of bronchial asthma and COPD.

Ipratropium bromide (IB) is a non-fat-soluble compound containing quaternary ammonium ions with a water solubility of 10 mg/ml (5). As a non-selective M-choline receptor blocker, it exerts bronchodilator effects by antagonising acetylcholine binding to M-choline receptors on bronchial smooth muscle (6). IB inhalation aerosol is a pressurised metered-dose aerosol unit that contains a solution of IB. It is a short-acting bronchodilator with the advantages of a small dose, fast onset and few adverse reactions. They are mainly used for preventing and treating COPD-related dyspnoea and mild to moderate bronchial asthma. It is one of the representative drugs of oral inhalation preparations (7). In order to evaluate the bioequivalence (BE) of OIDPs in terms of quality and efficacy, *in vitro* studies, human Pharmacokinetic (PK) studies and Pharmacodynamic (PD) studies are required (8). Human PK studies can directly reflect the speed and degree of drug entry into the systemic circulatory system and can tell the differences between preparations, even though they cannot explain the reasons for these differences. Its evaluation effectiveness has been recognised by the national drug regulatory agencies and has become an important part of being considered in the review and approval process (9, 10). The PBPK model established according to the drug’s physicochemical properties and physiological characteristics can be used to predict the PK behaviour of the drug *in vivo* and has been widely used to assist the development of oral products (11–13). However, there are few applications in the field of inhalation products. Although some research results have been achieved, there are limitations in the published literature on the division of lung deposition (14, 15). In this study, a PBPK model of IB inhalation aerosol was established in the B^2^O simulator (version 3.0, Shanghai Yinghan Pharmaceutical Technology Co., Ltd) to study the virtual bioequivalence of the test and reference products. The same simulator has already been used in bioequivalence studies (16–18), such as the study reported by Wu et al. 2022, the intranasal pharmacokinetic (PK) of the OC-01(varenicline) nasal spray was predicted using the nasal physiologically based pharmacokinetic (PBPK) model. The semi-PBPK model successfully depicted the absorption and distribution of intranasal varenicline in the respiratory tissues (17). The validated PBPK model can be used to simulate the deposition of IB in different lung regions after inhalation. It can also predict drug *in vivo* exposure under conditions such as with or without activated carbon blockade. Activated charcoal blocks the absorption of the drug from the gastrointestinal tract, allowing negligible drug entry into the systemic circulation *via* this pathway (19). The model reduced development time and cost through preparation design and process optimisation.

In the current study, a PBPK model was constructed and validated by integrating clinical data (Clinical study-1) and non-clinical data from an aerodynamic particle size distribution study into the B^2^O simulator. The model was used to assess different test and reference products’ bioequivalence. Results were compared to a second clinical study (Clinical study-2: registration number CTR20202291).

## 2. Materials and methods

### 2.1. Construction of the PBPK model and validation

#### 2.1.1. Construction of PBPK model

**Figure 1:**
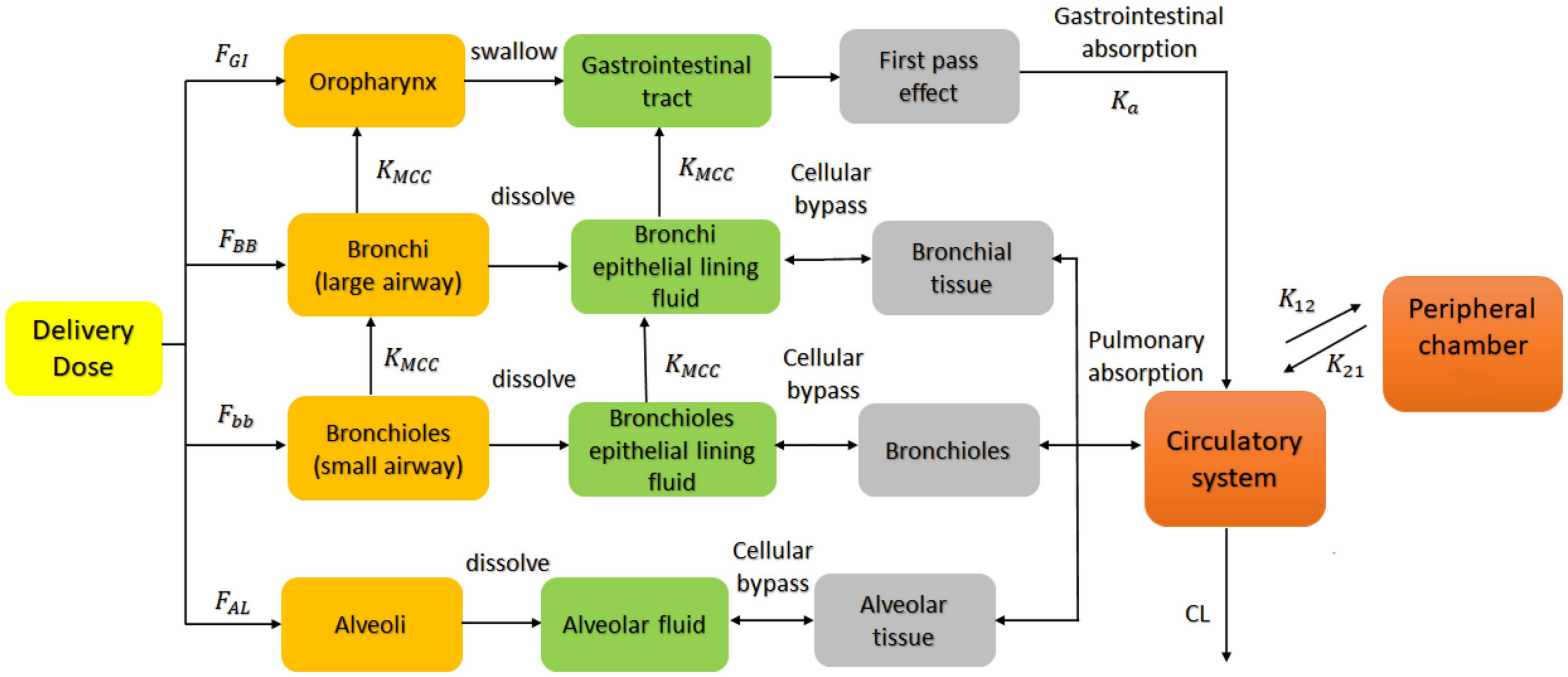
PBPK model describing the concentration-time profile of the ipratropium bromide (IB) inhalation aerosol. *F*_GI_, *F*_BB_, *F*_bb_ and *F*_AL_ are the percentage of drug deposited in the oropharynx, bronchi, bronchioles, and alveoli, respectively; *K*_MCC_ is the mucociliary clearance rate constant; *K*_a_ is the absorption rate constant; *K*_12_ and *K*_21_ the first-order distribution rate constants in the compartment model; CL the systemic clearance rate. This model is applied to the B^2^O simulator.

Drug lung and plasma exposure following inhalation is influenced by particle deposition, drug dissolution and drug absorption. Particle deposition largely depends on aerodynamic particle size and inhalation profiles such as flow rate and breath hold (20–22). Particles with size <=5 μm had a higher probability of bronchioles deposition, and particles <=2 μm more likely to deposit in alveolar lung regions. Ciliated epithelial cells clear insoluble particles from the central airways by mucociliary clearance (MCC), whereas deep lungs have large areas of thin alveolar cells without MCC (23). The PBPK model divided the human respiratory tract into four parts: the oropharynx, bronchi (large airways), bronchioles (small airways), and alveolar regions. Each site handles inhaled medication differently (Figure 1). We call the total amount of medicine entering the human body with airflow the ‘delivered dose’.

In this delivered dose, those particles larger than 9.0 μm were deposited directly in the oropharynx because of the inertial impact effect and then absorbed into the system by swallowing into the gastrointestinal tract; Particles equal to or between 5.8 and 9.0 μm were deposited in the bronchial area, mostly dissolved in the bronchial Epithelial Lining Fluid (ELF), and undissolved drug particles were expelled from the lungs into the oropharynx because of the Muco-Ciliary Clearance (MCC); Particles between 2.1 and 5.8 μm were deposited in the bronchioles, mostly dissolved in the bronchioles ELF, and a small part was excreted into the bronchi and bronchial ELF through MCC; Particles equal to or smaller than 2.1 μm entered the alveolar area and deposited and then dissolved in the alveolar fluid, unaffected by the clearance. These classifications are derived from the results reported by Cheng 2014 (24), in which particle deposition data were calculated for different regions of the respiratory system during 100% mouth breathing based on the ICRP model (25).

Drug particles dissolved in various lung regions were subsequently absorbed into the circulatory system. The model assumed that the model’s absorption, distribution, and elimination processes follow first-order kinetics, and a two-compartment model was used to describe the systemic disposition of the drug (14, 26, 27). The model also assumed that the ciliary clearance rate completely depends on the movement of the epithelial lining fluid with the cilia; that is, the drug in the epithelial lining fluid did not diffuse in the direction of the respiratory tract. Based on the individual differences of the population and the physiological parameters of healthy people (28, 29), the coefficient of variation of each parameter was implemented to be 30% (default value) in the B^2^O simulator. This simulator simulated the rate and extent of drug absorption using the advanced compartmental absorption and transit model (ACAT) (30). The ACAT model was developed based on the CAT model, in which a set of differential equations were used considering the simultaneous movement of a drug in solution through the gastrointestinal tract and the absorption of the dissolved material from each compartment into the portal vein (30).

In Figure 1, *F*_GI_, *F*_BB_, *F*_bb_, and *F*_AL_ are the percentages of drug deposited in the oropharynx, bronchi, bronchioles, and alveoli, respectively. These data are obtained from the aerodynamic particle size distribution (APSD) experiment. *K*_a_ is the gastrointestinal absorption rate constant. *V*_d_ is the apparent volume of distribution. CL is the system clearance rate. The parameters related to absorption *K*_a_, distribution *V*_d_, elimination processes CL and compartment model parameters, *K*_12_ and *K*_21_ were obtained from a preliminary study of Clinical study-1 (described below in Section 2.1.3), in which 12 subjects participated and completed the study. Other drug parameters such as *f*_up_, MW, and *F*_a_ were obtained from literature searches. All absorption and distribution processes in this model follow first-order kinetics. All the parameters and sources are listed in Table 1 and were used as input parameters in the B^2^O simulator.

**Table 1:** Main parameters used to establish the PBPK model of ipratropium bromide inhalation aerosol.

Parameter	Meaning	Value	Source
DD	Delivered dose	To be determined *in vitro*	APSD
*F*_GI_	Gastrointestinal tract deposited drug percentage	To be determined *in vitro*	APSD
*F*_BB_	Bronchial deposited drug percentage	To be determined *in vitro*	APSD
*F*_bb_	Bronchioles deposited drug percentage	To be determined *in vitro*	APSD
*F*_AL_	Alveolar area drug percentage	To be determined *in vitro*	APSD
*K*_a_	Absorption rate constant	0.7 h^−1^	Prior study
*V*_d_	Apparent volume of distribution	183.7 l	Prior study
CL	System clearance rate	53.3 l/h	Prior study
Compartment model parameters	First-order distribution rate constant	*K*_12_ = 8.645 h^−1^, *K*_21_ = 6.124 h^−1^	Prior study
*f*_up_	Plasma free fraction	0.91	DrugBank (31)
MW	Molecular weight (ipratropium)	332.5 g/mol	DrugBank (31)
*F*_a_	Absorption fraction	0.02	Pakes GE (32)
*K*_MCC_	Mucus-ciliary clearance rate constant	*K*_MCC_BB_ = 0.417 h^−1^; *K*_MCC_bb_ = 0.083 h^−1^	ICRP (25)

#### 2.1.2. Determination of aerodynamic particle size distribution of products

The APSD of the IB inhalation aerosol was determined using the Andersen Cascade Impactor (ACI) (8, 33). This determination occurs through the impaction and retention of particles on the collection plate present at each impactor stage (34). In the simulation, the drug content of the product at each level of ACI was calculated, respectively, and was input into the model as *in vitro* parameters for the simulation. According to the above-mentioned division method of particle size and deposition area, gastrointestinal deposition fraction (*F*_GI_) was calculated from the particles collected from the nozzle adapter to ACI level 1; bronchi deposition fraction (*F*_BB_) was calculated from the particles collected from ACI level 2 to 3; bronchioles deposition fraction (*F*_bb_) was calculated from particles collected from level 4 to 5; alveolar area deposition fraction (*F*_AL_) was calculated from particles collected from level 6 to 7.

Drug concentration was determined using the Liquid Chromatography–Mass Spectrometry (LC–MS) method, and the system mainly included a liquid chromatography (LC-30 AD, Shimadzu), a mass spectrometer (MS-8060, Shimadzu) and a chromatographic column (ACQUITY UPLC BEH C18, 2.1 mm × 50 mm, 1.7 μm, Waters). The mobile phase consisted of solvent A (100% in methanol) and solvent B (2 mM ammonium acetate and 0.025% acetic acid). The ratio of methanol/water was 85:15 (v/v). All reagents were purchased from Fisher Scientific and in chromatographic purity. The components were separated using gradient elution at a flow rate of 0.4 ml/min, a column temperature of 40°C and an injection volume of 10 μl. IB reference (lot 100,522–201,802, purity 95.8%, China Institute of Food and Drug test) was used to calculate drug concentration.

#### 2.1.3. Clinical study-1

A randomised, open-label, single-dose, cross-over clinical BE study under fasting conditions was conducted and registered with the National Medical Products Administration (NMPA), with registration number: CTR20201284. The study was approved by the Ethics Committee of Wuxi People’s Hospital Affiliated with Nanjing Medical University (approval number: LLPJ-I-15, 31st March 2020). The study was conducted in accordance with Good Clinical Practice regulations and the ethical principles in the Declaration of Helsinki. All the subjects signed the informed consent in person. They were trained to use the aerosol device correctly and complete the trial in accordance with the protocol. In protocol, subjects were asked to hold their breath for 10 s when inhaling the IB aerosol.

Forty healthy Chinese subjects with age >=18 years, male body weight >=50.0 kg, female body weight >=45.0 kg, and average body mass index of 19.0–26.0 kg/m^2^ were enrolled. According to medical history and physical examinations, all subjects were healthy and without significant diseases. Subjects were randomised into two groups using SAS software (version 9.4, SAS Institute). In each group, subjects inhaled 80 μg IB inhalation aerosol test product (*T*) or reference product (*R*) (20 μg/actuation throughout × 4 actuation) at four different periods with a 7-day washout time.

Eighteen blood samples under fasting condition (4 ml at each time point) were collected as follows: 0, 3, 6, 10, 15, 20, 25, 30, 45 min, 1, 2, 3, 4, 5, 6, 8, 12, 24 h. Samples were centrifuged at 2–8°C for 10 min, and the supernatants were collected and stored at −60°C. A validated LC–MS/MS method was used to determine the plasma concentrations (35). PK parameters were calculated using a non-compartment model in WinNonlin software (8.2 version, Pharsight). The meaning and calculation of PK parameters are listed in Table 2.

**Table 2:** The meaning of PK parameters from plasma concentration profiles.

PK parameter	Meaning
*C*_max_	Maximum plasma concentration in the plasma concentration-time profile
AUC_0−*t*_	Area under the plasma concentration-time curve from 0 to the last measurable time
AUC_0–∞_	Area under the plasma concentration-time curve from 0 to infinity
*T*_max_	the time to *C*_max_ in the plasma concentration-time profile
*T*_1/2_	Terminal elimination half-life; *T*_1/2_ = ln2/*λ_z_*
*λ_z_*	Elimination rate constants. Slope of a terminal end of the semi-log plasma concentration-time profile

#### 2.1.4. Verification of the PBPK model

When the APSD data were entered, the plasma concentration-time profiles of test and reference products were simulated. The formula for calculating the degree of fit *r*^2^ is described below (36):


(1) $$r^{2}=1-\frac{\sum_{i=1}^{n}{\left(C_{i}-\hat{C_{i}}\right)}^{2}}{\sum_{i=1}^{n}{\left(C_{i}-\bar{C_{i}}\right)}^{2}}$$

in which *C*_i_ is the measured concentration obtained from the human PK study, $\hat{C_{i}}$ is the predicted concentration simulated by the model, and $\bar{C_{i}}$ is the mean measured concentration. The closer *r*^2^ is to 1, the better the fitting degree between the model prediction and the observation (36). PE (percentage error) %, AFE (average fold error) and AAFE (absolute average fold error) are also used to evaluate the accuracy of the model, and the calculating formula is described below (37, 38):


(2) $$PE\%=\frac{\left|\bar{\mathrm{pred}}-\bar{obs}\right|}{\bar{obs}}\times 100\%$$

(3) $$AFE=10^{\frac{1}{n}\times \sum \mathrm{lg}\frac{\mathrm{pred}}{obs}}$$

(4) $$\mathrm{AAFE}=10^{\frac{1}{n}\times \sum |\mathrm{lg}\frac{\mathrm{pred}}{obs}|}$$

in which pred is the predicted value of the main PK parameter, $\bar{\mathrm{pred}}$ is the mean value; obs is the measured value of the main PK parameter, and $\bar{obs}$ is the mean value. When PE% < 20%, AFE < 2, AAFE < 3, the PBPK model could accurately predict the *in vivo* PK characteristics of the drug (37, 38). The chi-square test was also performed to compare test and reference products, with an alpha risk fixed at 5%.

### 2.2. PBPK modelling software

The PBPK model was implemented using the B^2^O simulator to predict drug exposure. It is a virtual drug development platform that integrates formulation development and other characteristic function modules, such as drug–drug interaction (DDI) and virtual bio-equivalence study (VBE). The geometric mean of all *C*_max_ and AUC*_t_* were calculated with lower and upper CI% (confidence interval) limits of 5–95%. If the geometric mean ratio of the PK parameters of the test and reference products fell within the equivalence interval (80–125%), the two products could be considered bioequivalent.

### 2.3. Carbon blocking using charcoal suspension

Carbon blocking treatment was used to study the effect of gastrointestinal tract absorption of IB inhalation aerosol on plasma concentration. 30 ml (approximately 3 g) of activated charcoal suspension was orally taken within 2 min before, immediately after administration, and 0.5 and 1 h after administration. Subjects were instructed to gargle and swallow the activated charcoal suspension, ensuring that the oral mucosa and upper gastrointestinal tract were completely covered with charcoal.

### 2.4. Clinical study-2

In Clinical study-2, subjects inhaled a different IB inhalation aerosol, and its bioequivalence with the reference product was studied. A randomised, open-label, single-dose, cross-over clinical BE study under fasting conditions was conducted and registered with the NMPA, with registration number: CTR20202291. The study was approved by the Ethics Committee of Wuxi People’s Hospital Affiliated with Nanjing Medical University (approval number: LLPJ-I-49). The study was conducted in accordance with Good Clinical Practice regulations and the ethical principles in the Declaration of Helsinki. All the subjects signed the informed consent in person. Subjects were trained to use the aerosol device correctly and complete the trial in accordance with the protocol. In protocol, subjects were asked to hold their breath for 10 s when inhaling the IB aerosol.

Thirty healthy Chinese subjects with age > 18 years, male body weight > = 50.0 kg, female body weight >=45.0 kg, and average body mass index of 19.0–26.0 kg/m^2^ were enrolled. According to medical history and physical examination, all subjects were healthy and without significant diseases. Subjects were randomised into two groups using SAS software (version 9.4, SAS Institute). In each group, subjects inhaled 80 μg IB inhalation aerosol test product (*T*) or reference product (*R*) (20 μg/actuation throughout × 4 actuation) at four different periods with a 7-day washout time.

Eighteen blood samples under fasting condition (4 ml at each time point) were collected as follows: 0, 3, 6, 10, 15, 20, 25, 30, 45 min, 1, 2, 3, 4, 5, 6, 8, 12, 24 h. Samples were centrifuged at 2–8°C for 10 min, and the supernatants were collected and stored at −60°C. A validated LC–MS/MS method was used to determine the plasma concentrations (35). PK parameters were calculated using a non-compartment model in WinNonlin software (8.2 version, Pharsight).

## 3. Results

### 3.1. Aerodynamic particle size distribution and deposition fractions

#### 3.1.1. Aerodynamic particle size distribution

The *in vitro* APSD of the IB inhalation aerosol test and reference products were measured using ACI, and the average deposition distribution of drug particles at each level per actuation (20 μg/actuation throughout) is shown in Table 3. After calculation, the median mass aerodynamic diameter (MMAD) values of both products were 1.14 ± 0.03 μm for the test product and 1.07 ± 0.05 μm for the reference product, and the geometric standard deviation (GSD) values were 3.09 ± 0.66 and 2.22 ± 0.30, respectively, (arithmetic means ±SD, *n* = 6).

**Table 3:** Determination of APSD data for ipratropium bromide (IB) inhalation aerosol test and reference products using ACI.

Stage	diameter/μm	Test/μg	Reference/μg
Inhaler device	NA^*^	3.23	3.14
Nozzle adapter	NA^*^	0.72	0.85
L-shaped connecting pipe	NA^*^	9.15	9.07
0 Stage	9.0	0.38	0.33
1 Stage	5.8	0.08	0.11
2 Stage	4.7	0.02	0.04
3 Stage	3.3	0.06	0.06
4 Stage	2.1	0.35	0.31
5 Stage	1.1	1.81	1.99
6 Stage	0.7	1.55	1.53
7 Stage	0.4	0.87	0.92
Filter paper layer	NA^*^	0.82	0.75
Delivered dose^**^	NA^*^	15.81	15.96

*NA: not applicable. **Delivery: the amount of drug deposited from the Nozzle adapter to the filter layer.

#### 3.1.2. Deposition fractions in different regions of the gastrointestinal tract and lungs

According to the *in vitro* APSD results and the deposition area classification, the deposition fractions of the test and reference product in the gastrointestinal tract and lung regions were calculated and shown in Table 4. It should be noted that all data for the deposition fractions in this paper refer to the percentage of the total dose (20 μg) in each segment.

**Table 4:** Ipratropium bromide (IB) inhalation aerosol deposition fractions for test and reference products.

Stage	Depositional area	Deposition fraction	Test	Reference
Nozzle adapter-Stage 1	Gastrointestinal tract	*F*_GI_	2.58%	2.59%
Stage 2—Stage 3	Bronchi	*F*_BB_	0.02%	0.03%
Stage 4—Stage 5	Bronchioles	*F*_bb_	0.54%	0.58%
Stage 6 to Stage 7	Alveolar area	*F*_AL_	12.10%	12.25%

### 3.2. Evaluation and verification of the PBPK model

#### 3.2.1. BE evaluation of clinical study

**Figure 2:**
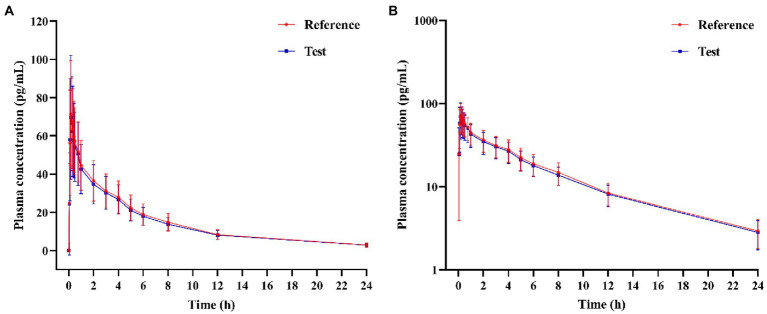
**(A)** Mean plasma concentration-time profiles, and **(B)** Semi-logarithmic plasma concentration-time profile of test and reference products after inhalation of 80 μg (20 μg/actuation × 4 actuation) of ipratropium bromide aerosol in healthy adult subjects under fasting condition.

In the PK study (Clinical study-1), two subjects withdrew from each group, and the remaining 38 subjects completed the trial according to the protocol. Chinese healthy adults inhaled 80 μg (20 μg/actuation × 4 actuation) IB aerosol test and reference products under fasting conditions; their mean plasma concentration-time and semi-logarithmic concentration-time profiles are shown in Figure 2. The result of BE analysis is displayed in Table 5, including *C*_max_, AUC_0−*t*_, AUC_0–∞_, *T*_1/2_, *T*_max_ and λ_z_. The observed plasma concentration of the test product was close to the reference product. The value of AUC_0−*t*_ of the reference product was 352.64 ± 95.10 h**·**pg./ml, and the test value (mean) was 336.50 ± 85.94 h**·**pg./ml. The *C*_max_ of the reference product was 78.71 ± 27.03 pg./ml, and the test value was 78.22 ± 32.30 pg./ml.

**Table 5:** PK parameters of the test and reference products after inhalation of 80 μg (20 μg/actuation × 4 actuation) of ipratropium bromide aerosol in Chinese healthy adult subjects under fasting condition.

PK parameter	Test (*n* = 38)	Reference (*n* = 38)
*C*_max_ (pg/ml)	78.22 ± 32.30	78.71 ± 27.03
AUC_0−*t*_ (h·pg./ml)	336.50 ± 85.94	352.64 ± 95.10
AUC_0–∞_ (h·pg./ml)	366.13 ± 92.94	381.54 ± 102.68
*T*_max_^*^(h)	0.17 (0.049, 1.000)	0.17 (0.099, 0.999)
*T*_1/2_ (h)	6.83 ± 1.38	6.62 ± 1.02
*λ_z_* (h^−1^)	0.105 ± 0.020	0.107 ± 0.017

*T_max_ is represented by median (minimum, maximum), and other parameters are represented by geometric mean (±standard deviation, SD).

A total of 11 adverse events occurred in eight subjects during the clinical study. All of the adverse events were grade one in severity (mild; asymptomatic or mild; clinical or diagnostic only; no treatment required). All were classified as ‘recovered’ without medical intervention, with no adverse events leading to subject withdrawal. Two subjects experienced multiple adverse events after using the reference product. The details of the events are listed in Table 6.

**Table 6:** Details of adverse events of Clinical study-1.

Adverse event	Test (*n* = 20)	Reference (*n* = 20)	Total
	Case	Case	Case
Elevated serum creatinine	4	1	5
Decreased neutrophil count	0	2	2
Decreased white blood cell count	0	2	2
Elevated blood bilirubin	0	1	1
Administration fear	1	0	1
Total	5	6	11

#### 3.2.2. Verification of the PBPK model

**Figure 3:**
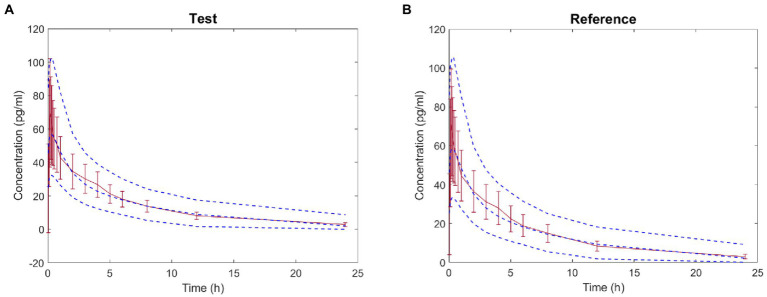
Comparison between the predicted plasma concentration-time profile of ipratropium bromide (IB) inhalation aerosol (blue dotted line) and the observations from Clinical study-1 (mean ± SD and red trend line; A: test product; B: reference product).

The virtual BE study in healthy subjects after inhalation of IB aerosol was performed in the B^2^O simulator, with the parameters listed in Tables 1, 4. The comparison of simulated and observed PK profiles of test and reference products from Clinical study-1 are shown in Figure 3. The model prediction and observation fit degree *r*^2^ of the test and reference product were 0.9837 and 0.9846, respectively. The predicted and observed values of the main PK parameters *C*_max_, AUC_0−*t*_ and AUC_0–∞_ were compared, and PE%, AFE and AAFE were calculated. The results are shown in Table 7. Both PE% values were smaller than 20%, AFE values smaller than two, and AAFE values smaller than three. The value of (*X*)^2^ was calculated to be 4.3%. These verification results suggested that the PBPK model reasonably predicted *in vivo* performance of IB aerosol.

**Table 7:** Comparison of predicted and observed values of main PK parameters of the test and reference products.

Product	Main PK parameter	PE%	AFE	AAFE
Test	*C*_max_	4.07	0.93	1.39
	AUC_0−*t*_	1.46	1.02	1.22
	AUC_0–∞_	3.55	0.96	1.21
Reference	*C*_max_	4.55	0.93	1.37
	AUC_0−*t*_	4.33	1.00	1.26
	AUC_0–∞_	10.12	0.94	1.28

### 3.3. Simulation of deposition of IB inhalation aerosol in different regions of the lung after inhalation

**Figure 4:**
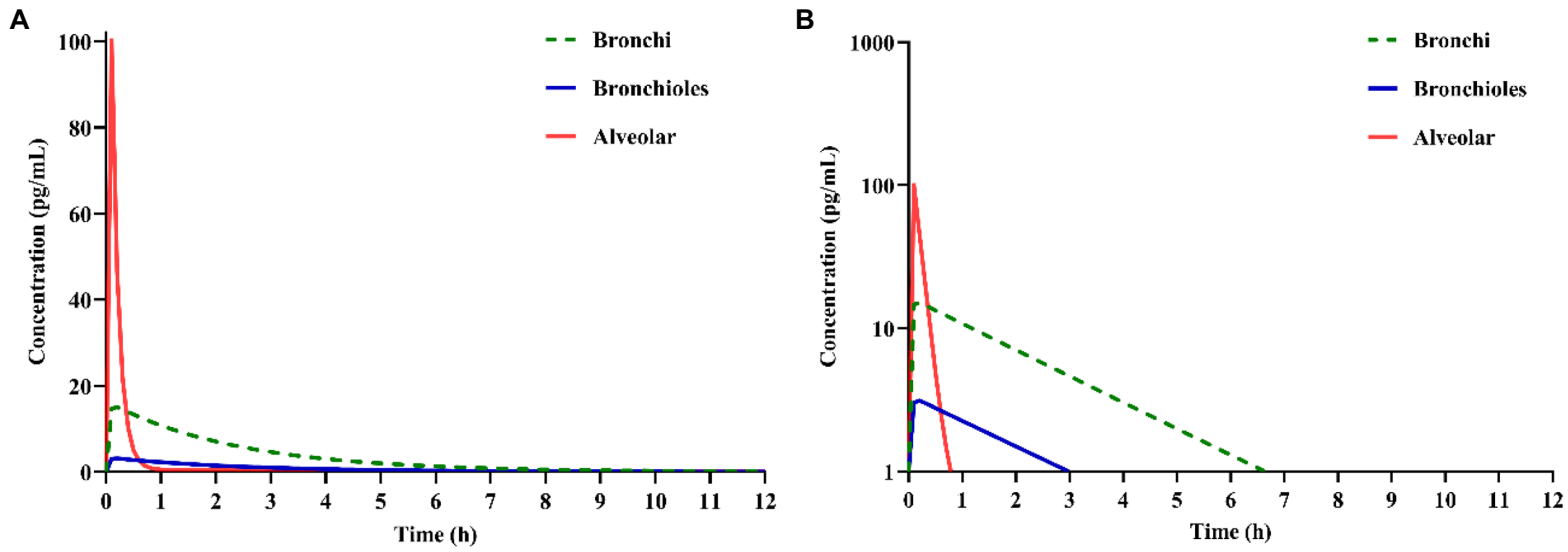
Simulation of ipratropium bromide (IB) inhalation aerosol **(A)** concentration-time curves, **(B)** semi-logarithmic concentration-time curves in different lung regions.

With the validated parameters in the PBPK model, the deposition of IB inhalation aerosol (reference product) in different lung regions was simulated. The drug concentration-time curves in bronchi, bronchioles and alveoli are shown in Figure 4. From this figure, we can see that most of the particles accumulated in the alveolar region immediately after inhalation and were cleared within an hour. Less than 20% of the particles accumulated in the bronchi region after inhalation and were cleared within 7 h. A small part of the particles accumulated in the bronchioles region after inhalation and were cleared within 3 h.

### 3.4. Effect of gastrointestinal tract absorption of ipratropium bromide inhalation aerosol on plasma concentration

**Figure 5:**
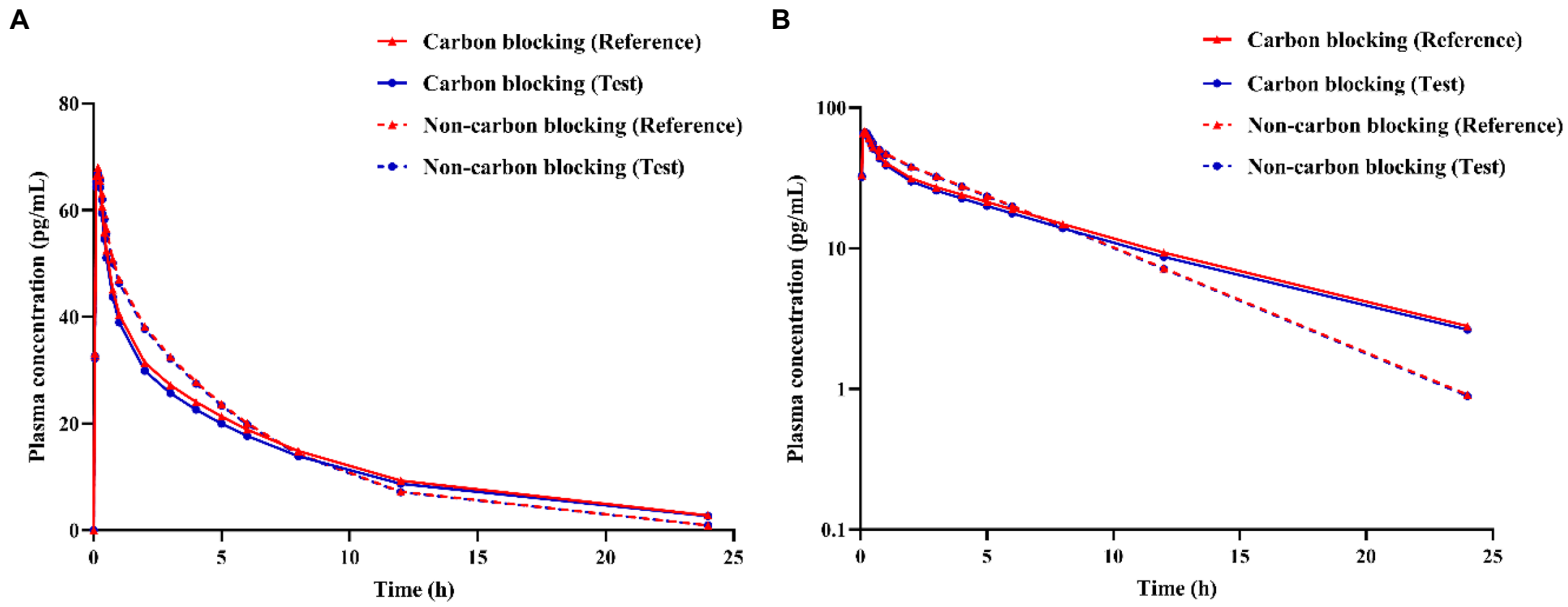
Simulation of ipratropium bromide inhalation aerosol **(A)** concentration-time curves, **(B)** semi-logarithmic concentration-time curves under different conditions (carbon blocking/ non-carbon blocking).

Activated charcoal was used to block the absorption of the drug from the gastrointestinal tract. It can be seen from Figure 5 that the plasma concentration of the test product under carbon blocking and non-carbon blocking conditions are relatively close, indicating that the effect of the gastrointestinal tract absorption of IB inhalation aerosol on plasma concentration could be negligible. Like the reference product, the effect of gastrointestinal absorption on plasma concentration could be negligible.

### 3.5. Prediction of bioequivalence of ipratropium bromide aerosol product with different APSD in Clinical study-2

#### 3.5.1. Aerodynamic particle size distribution and deposition fractions

According to the *in vitro* APSD results and the deposition area classification, the deposition fractions of the test and reference products in the gastrointestinal tract and lung regions were calculated and shown in Table 8. The deposition fraction refers to the percentage of the total dose of 20 μg in each segment.

**Table 8:** The deposition fraction of ipratropium bromide inhalation aerosol for test and reference products used in Clinical study-2.

Stage	Depositional area	Sedimentation fraction	Test	Reference
Nozzle adapter-Stage 1	Gastrointestinal tract	*F*_GI_	2.44%	2.28%
Stage 2—Stage 3	Bronchi	*F*_BB_	0.03%	0.02%
Stage 4—Stage 5	Bronchioles	*F*_bb_	0.52%	0.55%
Stage 6 to Stage 7	Alveolar area	*F*_AL_	11.10%	11.50%

#### 3.5.2. BE evaluation and comparison with Clinical study-2

**Figure 6:**
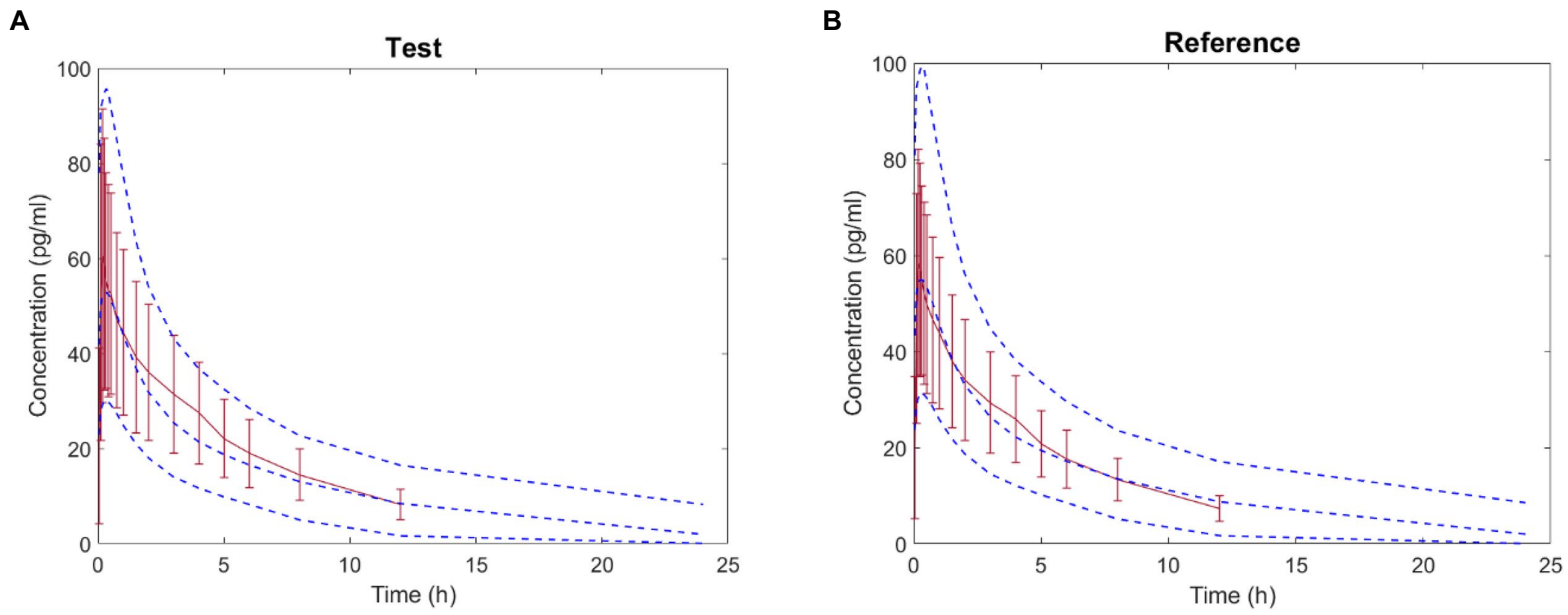
Comparison between the predicted plasma concentration-time profile of ipratropium bromide inhalation aerosol (blue dotted line) and the observations in Clinical study-2 (mean ± SD and red trend line; **A**: test product; **B**: reference product).

Based on the parameters (*V*_d_, CL, *K*_12_, *K*_21_) and *in vitro* APSD data, the virtual BE study in healthy subjects after inhalation of products in Clinical study-2 was performed in the B^2^O simulator. The comparison of simulated and observed PK profiles of both products is shown in Figure 6. These results suggested that the plasma concentration of the test product was close to those of the reference product. In Table 9, parameters are presented by geometric mean. *T* represented the test product, and *R* represented the reference product. The ratios (*T*/*R*) of the main parameters *C*_max_, AUC_0−*t*_ and AUC_0–∞_ were 99.76, 104.3, and 105.3%, with 90% confidence intervals (CI) of 92.91–107.1%, 98.3–110.6%, and 99.7–111.3%, respectively, indicating that the test product was bioequivalent to the reference product. This result was consistent with the result from Clinical study-2.

**Table 9:** The mean ratio (*T*/*R*) and 90% confidence of main PK parameters (*C*_max_, AUC_0−*t*_ and AUC_0–∞_) after inhalation of product in Clinical study-2 in healthy subjects under fasting condition.

PK parameter	*T* (*n* = 300)	*R* (*n* = 300)	Geometric mean (*T*/*R*) %	90% CI
*C*_max_	60.66	60.81	99.76	92.91–107.1
AUC_0−*t*_	312.0	299.2	104.3	98.3–110.6
AUC_0–∞_	338.9	321.7	105.3	99.7–111.3

**Figure 7:**
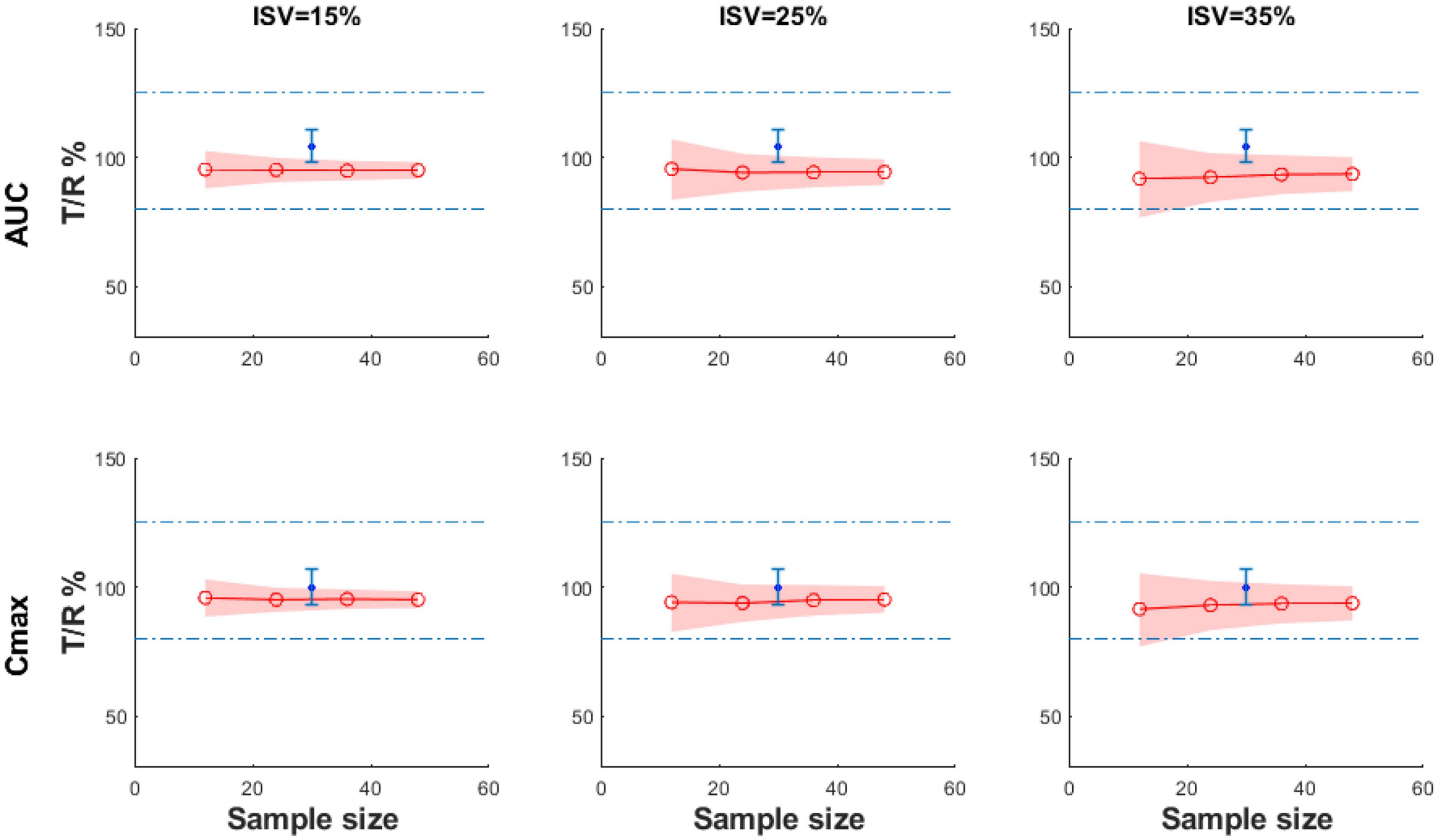
The BE result of the virtual test product compared with the reference product with different sample sizes and ISV% under fasting conditions (the blue dot: the geometric mean of the observed *T*/*R* ratio; the blue error bars: the 90% confidence interval of observed *T*/*R* ratio; the red line: the geometric mean value of *C*_max_ *T*/*R*% or AUC_0−*t*_ *T*/*R*%; the shadow: 90% CI).

In order to find the effects of intra-subject variability (ISV) and sample size on the bioequivalent study, the virtual simulations with different ISV values (ISV% = 15, 25 and 35%) and different sample sizes (*n* = 12, 24, 48) were performed, and the results are shown in Figure 7. ISV is related to the response time and has been defined as reaction time standard deviation. The *y*-axis is the ratio of AUC (*T*/*R*) or *C*_max_ (*T*/*R*), and the shading represents the 90% CI of the ratio. According to BE guidance, the ratio of AUC (*T*/*R*) % or *C*_max_ (*T*/*R*) % should be within the range of 80–125% (39). When the shading narrows, it indicates that the test and the reference products are more likely to be bioequivalent. In these figures, the shading narrows as the ISV decreases from 35 to 15%, indicating that the test and reference products are more likely to be bioequivalent if the results are tested from a population of subjects with less variability. In addition, even if the results are similar, if the test and reference products are tested in a larger sample size, the products are more likely to be bioequivalent.

## 4. Discussion

The overall exposure of OIDPs in the lungs is complex, involving processes such as local drug deposition and dissolution, transepithelial membrane transport, and pulmonary physiologic clearance. The aerosol containing IB enters the respiratory tract with airflow and is deposited on the surface of epithelial cells at all bronchi/bronchioles and alveoli levels through inertial impact, gravitational sedimentation and Brownian motion. Solubilised IB is absorbed into tissues mainly *via* the alternative cellular pathway (26). In the alveoli, the IB delivered to the alveoli is rapidly dissolved and absorbed into the blood due to its large surface area, wide gas-blood exchange interface, and relatively thin alveolar walls, even in the presence of slower-clearing macrophage phagocytic mechanisms (14, 40).

In the study reported by Cheng 2014, the human respiratory tract can be divided into three anatomical regions, including the extra thoracic region, the trachea-bronchial region and the pulmonary region (24). The extra thoracic region includes the nasopharyngeal region, the tracheobronchial tree includes the trachea and 16 generations of branching airways, and the pulmonary region includes alveolar ducts and alveolar sacs (24). According to this division, the respiratory tract in this PBPK model was divided into the oropharyngeal-gastrointestinal tract, bronchi, bronchioles, and alveolar regions. *In vitro* techniques such as cascade impactors can be used to measure the particle size distribution of an inhaled medication, and this information can be combined with inhalation profiles such as flow rate and breath hold to predict the regional lung deposition of the drug (20–22). Flow rate and breath hold were not considered to simplify the model. The deposition fractions of particles in each area were calculated, respectively, as input parameters in the B^2^O simulator. Combined with the physicochemical parameters of IB, the PK profile of the drug was generated and validated with the clinical study (Clinical study-1). Parameters were saved in the model for further prediction.

It is generally believed that drug particles <0.5 μm may be exhaled because residence time is too short for depositing (41). However, when the breath-hold time was extended to 10 s, the residence time of fine particles in the lung increased, and the deposition rate was significantly improved (42). In this clinical study, subjects were asked to hold their breath for 10 s when inhaling the IB aerosol.

Exposure to OIDPs depends on deposition and the regional distribution of the drug in the lungs. In this study, the time-varying distribution of the drug in each region was simulated using the established PBPK model, providing additional evidence for BE assessment. PK studies can reflect the exposure of OIDPs absorbed into the blood through the gastrointestinal tract and lungs after inhalation and can be used for the safety evaluation of products. In cases when drugs have low oral bioavailability and a negligible effect of gastrointestinal absorption on systemic exposure, PK studies can be used to assess both products’ bioequivalence (43). IB has low bioavailability of 0.03–6.9% and a low absorption fraction of 0.02 (44). Absorption rate has little effect on PK exposure (45).

The European Medicines Association (EMA) recommends using activated charcoal to block absorption from the gastrointestinal tract, with negligible drug entry into the systemic circulation *via* this route (46). This study simulated IB inhalation aerosol concentrations with or without carbon blocking. The results indicated that the drug had a negligible effect on gastrointestinal absorption on systemic exposure, and PK studies can be used to assess the bioequivalence of test and reference products. In the future, the *in vivo* PK results of different doses and batches of IB inhalation aerosols from different companies can be simulated to identify the key attributes that affect product bioequivalence. The simulation with bronchial asthma or COPD population can also predict the PK characteristics of the product in the patient population, thereby shortening the research and development cycle and saving research and development costs.

Limitations of this PBPK model include the following: the model does not consider the effects of lung transporters and metabolic enzymes; inhalation profiles such as flow rate and breath hold were not considered; the accuracy of the PBPK model depends on the accuracy of the parameters, which need to be continuously optimised and adjusted; the ACI device specified in the Pharmacopoeia to measure the APSD of drug particles is a standard method for evaluating product quality, but it may lack physiological significance, for example, the L-shaped connecting tube cannot simulate the mouth and throat, and constant flow rate cannot represent actual breathing. In the future, artificial throats and artificial lungs with actual breathing profiles can be used to determine more accurate deposition distribution; drug concentration-time curves in different lung regions (bronchioles, bronchioles and alveoli) were obtained by model simulations and could not be validated with anthropometric data.

## 5. Conclusion

This PBPK model provided a reliable method for exploring the correlation between *in vitro* APSD and *in vivo* PK studies of IB aerosols. The model study helps guide the design and optimisation of inhaled doses and devices and provides scientific support for accelerating the development, review and approval of inhaled drug products with significant clinical efficacy. The simulation results indicated that the test and reference products were bioequivalent.

## Data availability statement

The datasets presented in this article are not readily available because the data supporting this study’s findings are available from the corresponding author upon reasonable request. Requests to access the datasets should be directed to heqing0510@163.com.

## Ethics statement

The study was conducted and registered with the National Medical Products Administration (NMPA), with registration number: CTR20201284. The study was approved by the Ethics Committee of Wuxi People’s Hospital Affiliated with Nanjing Medical University (approval number: LLPJ-I-15, 31th March 2020). The patients/participants provided their written informed consent to participate in this study.

## Author contributions

QH: conceptualization. JZ: data curation, methodology, and wring-original draft. KW: formal analysis and software. BL: investigation. QH: project administration. XL, SH, and JL: writing-review and editing. XY: visualization. All authors contributed to the article and approved the submitted version.

## Conflict of interest

KW, XL, XY, and JL were employed by Yinghan Pharmaceutical Technology (Shanghai) Co., Ltd.

SH was employed by Sichuan Purity Medical Technology Co., Ltd., China during the study period.

The remaining authors declare that the research was conducted in the absence of any commercial or financial relationships that could be construed as a potential conflict of interest.

## Publisher’s note

All claims expressed in this article are solely those of the authors and do not necessarily represent those of their affiliated organizations, or those of the publisher, the editors and the reviewers. Any product that may be evaluated in this article, or claim that may be made by its manufacturer, is not guaranteed or endorsed by the publisher.
